# Effects of Probiotics Supplementation on CRP, IL-6, and Length of ICU Stay in Traumatic Brain Injuries and Multiple Trauma Patients: A Systematic Review and Meta-Analysis of Randomized Controlled Trials

**DOI:** 10.1155/2022/4674000

**Published:** 2022-12-05

**Authors:** Nooshin Noshadi, Marzieh Heidari, Mohammad Naemi Kermanshahi, Meysam Zarezadeh, Sarvin Sanaie, Mehrangiz Ebrahimi-Mameghani

**Affiliations:** ^1^Department of Clinical Nutrition, Faculty of Nutrition & Food Sciences, Tabriz University of Medical Science, Tabriz, Iran; ^2^Research Center for Integrative Medicine in Aging, Aging Research Institute, Tabriz University of Medical Sciences, Tabriz, Iran; ^3^Nutrition Research Center, Department of Biochemistry and Diet Therapy, Faculty of Nutrition & Food Sciences, Tabriz University of Medical Science, Tabriz, Iran

## Abstract

**Method:**

This meta-analysis aims to evaluate the effectiveness of probiotics in reducing inflammatory biomarkers and the length of intensive care unit (ICU) stays. PubMed-Medline, SCOPUS, Embase, and Google Scholar databases up to July 2021 were searched. The meta-analysis was carried out using random-effect analysis. To determine the sources of heterogeneity, subgroup analyses were performed. In case of the presence of publication bias, trim and fill analysis was carried out. The Cochrane Collaboration tool was used for checking the quality assessment. We hypothesized that probiotics would improve inflammatory markers (CRP and IL-6) and the length of ICU stay in traumatic brain injury and multiple trauma patients.

**Results:**

The present meta-analysis, which includes a total of seven studies, showed that there were no significant effects of probiotics supplementation on interleukin (IL)-6 (Hedges's *g* = −2.46 pg/ml; 95% CI: −12.16, 7.25; *P*=0.39), C-reactive protein (CRP) (Hedges's *g* = −1.10 mg/L; 95% CI: −2.27, 0.06; *P*=0.06), and the length of staying in ICU. The overall number of RCTs included in the analysis and the total sample size were insufficient to make firm conclusions.

**Conclusion:**

As a result, more carefully designed RCTs are needed to investigate the effect of probiotics on inflammatory biomarkers and the length of ICU stay in traumatic brain injuries and multiple trauma patients in greater detail.

## 1. Introduction

Traumatic injuries are the leading cause of morbidity and death in young people, and the incidence of trauma in patients with serious injuries is increasing [1–3]. The incidence rate of TBI is generally higher in developing nations than in more developed countries, and by 2030, it is expected to surpass several diseases as the leading cause of death and disability. For instance, the TBI incidence rate in Asia is 344 per 100,000 [4]. Nearly 80% of trauma deaths are caused by hemorrhage, central nervous system damage, or a combination of both [5]. Multiple immunological dysfunctions and metabolic changes are the subsequent life-threatening conditions of multiple traumas [6]. Immune system dysfunction is directly related to an increased risk of infection in trauma patients. Increased infectious complications in critically ill patients due to immune response impairment result in a higher mortality rate [7, 8]. Traumatic brain injury (TBI) is associated with immune system dysfunction and is often the result of a collision with a motor vehicle, violence, or a fall [9–11]. One of the more valuable therapeutic agents to decrease illnesses triggered by oxidative stress is natural compound-based antioxidants because they play a protective role in preventing the generation of free radicals [12, 13]. Recently, the importance of diet and antioxidants and complementary therapies in critically ill patients has been identified [14]. Several clinical studies have examined the effect of antioxidant micronutrients as monotherapy or combination treatment as a component of an antioxidant strategy for critically ill systemic inflammatory response syndrome (SIRS) patients [15, 16]. The results of recent studies show that in critically ill patients, the most severe cases of SIRS are associated with the most severe antioxidant reduction [17].

Probiotics are living bacteria that provide health benefits to the host when administered in sufficient amounts [18–20]. Prebiotics are foods or supplements that include nonliving, indigestible fibers that selectively increase the growth and/or activity of beneficial indigenous probiotic bacteria [21]. Many clinical trials and meta-analysis efforts have focused on the role of reducing ventilator-associated pneumonia. Probiotics appear to decrease infectious complications, including ventilator-associated pneumonia, and may influence the intensive care unit mortality [22]. Recent studies on probiotic use in critical care have focused on a range of outcomes after critical illness [23]. In a study performed in 2017, the consumption of probiotic yogurt showed beneficial effects on blood glucose, glycated hemoglobin, blood pressure, and serum lipid levels [20]. Synbiotics derived from a mix of prebiotics and probiotics function synergistically to support healthy gut flora. Mixed-strain microorganisms may have a more substantial impact on the gut microbiota [24]. Several mechanisms, such as immunoglobulin A production and mucus stimulation, suppression of nuclear factor kappa B (NF-kB) activation in epithelial cells, anti-oxidative effects, liberation of antimicrobial factors, and alteration of intestinal flora by inducing antimicrobial peptides in host cells, have been proposed for the benefits of probiotics [25–27].

Recently, there has been a great deal of interest in the use of probiotics to decrease inflammatory factors and oxidative stress markers [28]. It has been shown that probiotics suppress the expression of inflammatory cytokines such as NF-kB, interleukin (IL)-6, IL-10, C-reactive protein (CRP), and tumor necrosis factor-alpha (TNF-*α*) [29, 30]. Inflammation rapidly increases due to immunological disruption, which may increase the length of an intensive care unit (ICU) stay following trauma [31, 32].

The length of ICU and hospital stays was significantly reduced after consuming probiotics such as Lactobacillus, Bifidobacterium, and Streptococcus [33]. Based on a recent meta-analysis in 2017, probiotic administration can considerably decrease serum/plasma CRP levels [29]. Furthermore, another meta-analysis carried out in 2019 demonstrates that probiotic supplementation in critically ill patients could significantly reduce serum CRP levels [34]. Additionally, in 2017, a meta-analysis showed that prebiotic and synbiotic supplementation is associated with decreased serum CRP levels [35].

Several studies have demonstrated the potential effects of probiotics on inflammation in different diseases [36–38]. Furthermore, several clinical trials have investigated the effects of probiotic supplementation on inflammatory biomarkers and the length of stay in patients with brain trauma and multiple traumas; the results have been controversial. As a result, we conducted this study, and we hypothesized that probiotics would improve inflammatory markers (CRP and IL-6) and the length of ICU stay in traumatic brain injury and multiple trauma patients. Therefore, in this systematic review and meta-analysis, we searched different databases for published randomized control trials (RCTs) to analyze the impact of probiotic intake on the levels of inflammatory markers (CRP and IL-6) and the length of ICU stays.

## 2. Methods

### 2.1. Search Strategy

The Preferred Reporting Items for Systematic Reviews and Meta-analysis (PRISMA) statement was planned, conducted, and reported in the meta-analysis [39]. Eligible RCTs were identified using Scopus, PubMed, Embase, and Google Scholar databases for English-language publications from the inception dates to July 2021. Supplementary Table 1 provides details of the search terms. We used a snowball sampling approach to scan the citation lists of retrieved articles for additional articles relevant to the research topics and retrieved relevant titles. Additionally, to improve the sensitivity of our search strategy, the wild-card term “^*∗*^” was implied.

### 2.2. Study Selection Criteria, as Well as Inclusion and Exclusion Criteria

The following inclusion criteria were used to select trials: (1) published in the English language; (2) randomized placebo-controlled trials with a parallel or cross-over design; (3) RCTs comparing probiotics or synbiotic supplements to a placebo or no intervention group; and (4) patients with trauma and brain injuries.

Exclusion criteria included the following: (1) articles without a placebo or treatment group; (2) observational articles; (3) in vitro and in vivo studies, letters, conference abstracts, reviews, or case reports; and (4) inadequate data on the baseline or end-of-trial.

### 2.3. Data Extraction

Two independent researchers (NN and MH) extracted the following data: first author's name, year of publication, country of origin, study design, type of probiotic microbes, number of patients, age, gender, dose, and duration of supplementation. Any discrepancies were discussed and resolved with a third author (MZ).

### 2.4. Quality Assessment

The methodological quality of included studies was independently evaluated by two researchers (NN and MH) using the Cochrane risk of bias criteria: random sequence generation, allocation concealment, personnel's, assessors', and participants' blinding, incomplete outcome data, selective outcome reporting, and other possible causes of bias [40]. Each quality item was stratified as low risk, high risk, or unclear risk of bias.

### 2.5. Summary of Evidence: Grade Criteria

According to the Cochrane Handbook for systematic reviews of interventions, we used the GRADE approach to evaluate the overall quality of the evidence. Each outcome was evaluated using five criteria: (1) bias risk, (2) consistency, (3) directness, (4) precision, and (5) publication bias [41]. Four quality levels were determined using the GRADE test: high, moderate, low, and very low. The quality declined by one level when each factor was not met [42].

### 2.6. Statistical Analysis

The mean differences and standard errors of serum/plasma CRP, IL-6, and stay in ICU between probiotic, synbiotic, and control groups were used to calculate the overall effect sizes. A standard deviation (SD) was determined using the following formula when a standard error (SE) was given instead of SD: SD = SEM × square root (*n*), where “*n*” is the number of samples per group [43]. The random-effect model for the standardized mean difference was used in all analyses. Random-effect models consider different sources of uncertainties, including within-study (sampling or estimation) error and between-study variance, while fixed-effect models take only the within-study error into account. Therefore, a random-effect model can provide more conservative results than a fixed-effect model. The effect size was estimated based on Hedges's *g* for staying in the ICU and serum/plasma levels of CRP and IL-6. Due to the small number of included studies, the Hartung–Knapp adjustment was performed. The heterogeneity of included studies was assessed using Cochran's *Q* test and *I*^2^ statistic [44]. The *I*^2^ index *I*^2^ > 50% was deemed remarkable for heterogeneity. To investigate possible sources of heterogeneity, subgroup analysis was performed according to the duration of intervention and patients' age for the CRP factor. However, subgroup analysis was performed based on age for stays in ICU. Egger's regression test was used to assess the presence of publication bias [45]. Statistical analysis was performed using Stata software, version 16 (Stata-Corp., College Station, TX, USA). *P* value of less than 0.05 was recognized as statistically significant.

## 3. Results

### 3.1. Study Selection

**Figure 1:**
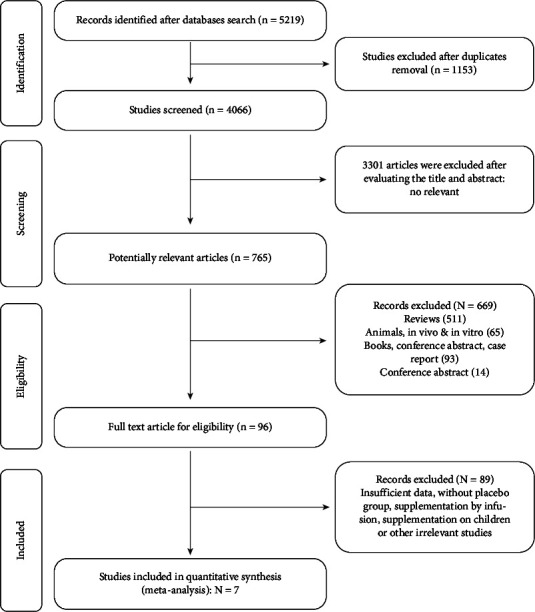
Flowchart of study selection.

The initial search yielded 5219 results. After removing duplicates, 4066 titles and abstracts were screened for possibly relevant RCTs. Finally, seven studies were enrolled in the meta-analysis following the evaluation of these studies. The strength of agreement using the kappa coefficient measures the agreement between two reviewers. In this study, kappa statistics were approximately 0.80 (Figure 1). presents the articles included in this review. The characteristics of the studies included in Table 1 were published between 2006 and 2020.

### 3.2. Study Characteristics

There were five studies that assessed serum/plasma levels of CRP, three for IL-6, and four studies on staying in the ICU. In the present meta-analysis, a total of 413 participants were included. These samples consisted of trauma patients and traumatic brain injuries. The duration of the intervention ranged from 7 to 56 days.

### 3.3. Risk of Bias Assessment

**Figure 2:**
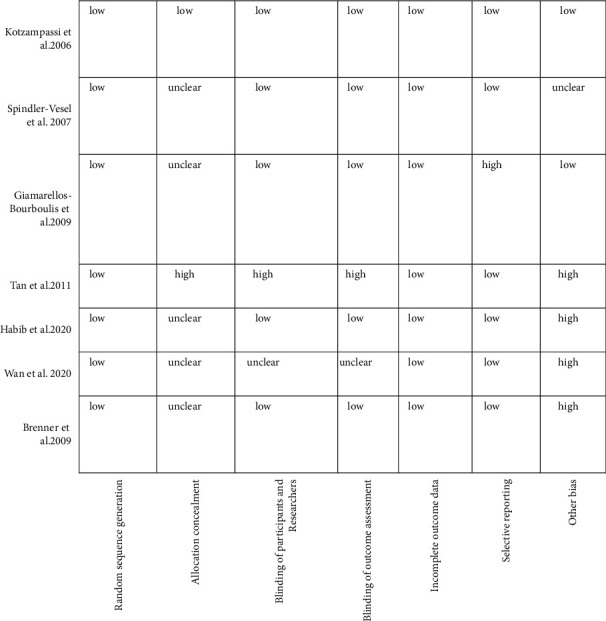
Quality of bias assessment of included studies according to the Cochrane guidelines.

The Cochrane collaboration's parameters were examined by two reviewers, and they reached 100% agreement. All of the studies contained sufficient information about random sequence generation. Most of the studies reported inadequate information about allocation concealment [30, 46–50]. However, the majority of the studies reported adequate information about the blinding of participants and researchers and the blinding of outcome assessment [47–51]. Selective reporting was at a high risk of bias only in one trial [47]. Incomplete outcome data in all of the studies showed a low risk of bias.  Figure 2 shows the quality of the bias assessment in detail.

### 3.4. Effects of Probiotic/Synbiotic on CRP

**Figure 3:**
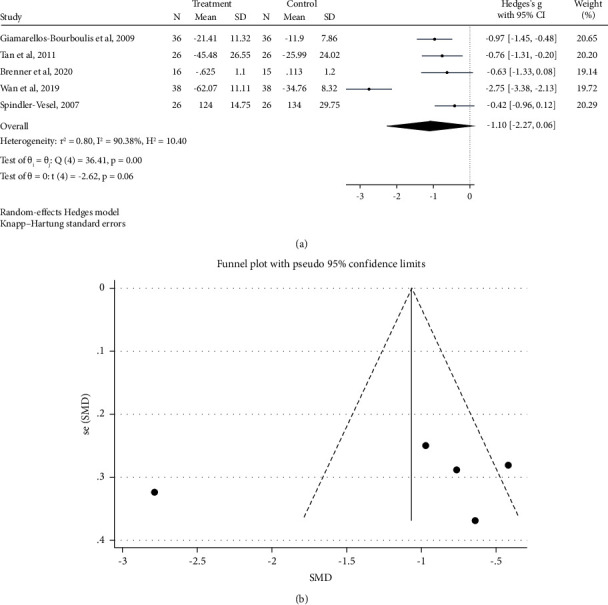
Forest plot and funnel plot of randomized trials investigating the effects of probiotic supplementation on CRP levels.

Figure 3(a) shows a forest plot of the pooled effect of probiotic/synbiotic consumption on plasma CRP levels. There were seven trials with 413 patients that compared serum/plasma CRP levels between the control and intervention groups. Probiotic/synbiotic supplementation had no significant effect on plasma levels of CRP (Hedges's *g* = −1.10 mg/L; 95% CI: −2.27, 0.06; *P*=0.06).

The between-study heterogeneity was considerable (*I*^2^ = 90.38%, *P* < 0.001), that age and type of supplement were identified as the sources of it (Table 2). There was a significant decrease in serum/plasma CRP in the subgroup of patients with a mean age of >40 years (SMD = −0.742 mg/L; 95% CI: −1.061, −0.423; *P* < 0.001) and duration of intervention ≥21 days (SMD = −0.722 mg/L; 95% CI: −1.166, −0.277; *P*=0.001).

In the sensitivity analysis, the exclusion of any single study did not affect the overall estimate for the impact of probiotic/synbiotic supplementation on serum/plasma CRP concentrations (95% CI: −1.895, −0.334). The CRP funnel plot supports the existence of a publication bias (Figure 3(b)). However, no evidence of publication bias was found using Egger's test (*P*=0.672). The overall quality of CRP evidence was moderate, which was further declined by inconsistency based on the GRADE approach (Table 3).

### 3.5. Effects of Probiotic/Synbiotic on IL_6

**Figure 4:**
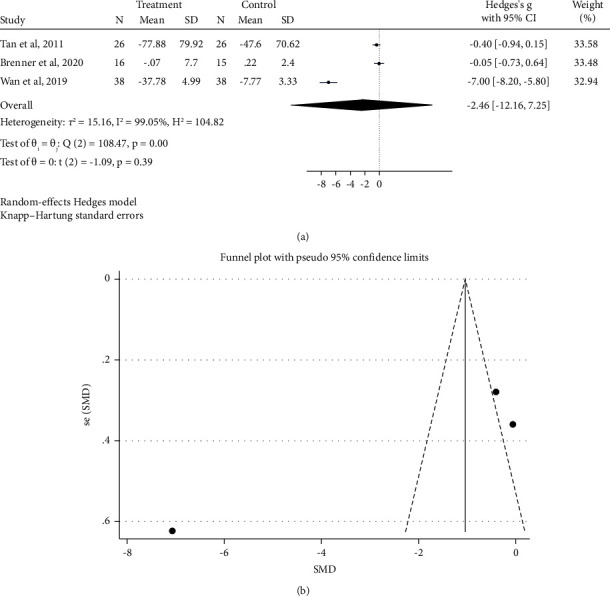
Forest plot and funnel plot of randomized trials investigating the effects of probiotic supplementation on IL-6 levels.

The results of four articles with 266 patients indicated that probiotic/synbiotic supplementation had no significant effect on plasma levels of IL-6 (Hedges's *g* = −2.46 pg/ml; 95% CI: −12.16, 7.25; *P*=0.39) (Figure 4(a)).

Removing any single study in the sensitivity analysis showed no effect on the overall estimate for the impact of probiotic/synbiotic supplementation on plasma IL-6 concentrations (95% CI: −5.682, 0.770). According to the GRADE approach, the total quality of the evidence for IL-6 was considered low because of inconsistency and imprecision (Table 3).

### 3.6. Effects of Probiotic/Synbiotic on the Length of Staying in ICU

**Figure 5:**
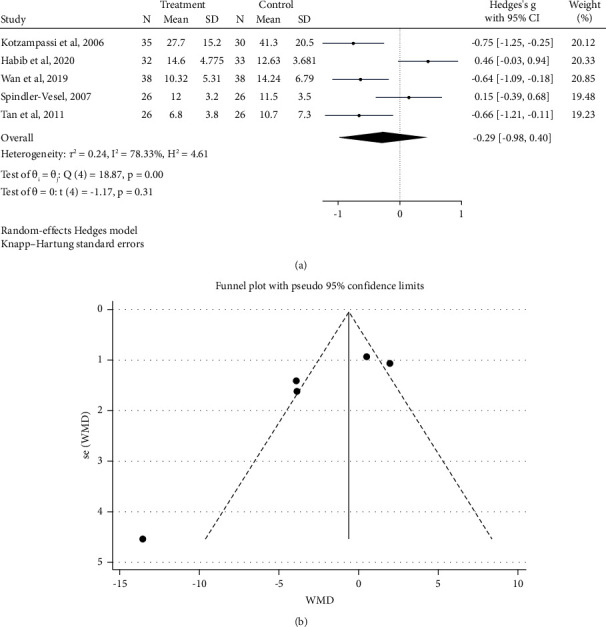
Forest plot and funnel plot of randomized trials investigating the effects of probiotic supplementation on the length of ICU stays.

Figure 4 indicates the forest plot of the pooled effect of probiotic/synbiotic intake on the length of stay in the ICU. The result of four our studies which included 258 patients demonstrated that probiotic/synbiotic supplementation had no significant effect on the length of staying in ICU (Hedges's *g* = −0.29 days; 95% CI: −0.98, 0.40; *P*=0.31) (Figure 5(a)).

There was evidence of significant between-study heterogeneity (*I*^2^ = 78.33%, *P* < 0.001). There were no significant effects of probiotic/synbiotic on the length of stay in ICU after subgroup analysis by mean age.

Removing any single study in the sensitivity analysis did not affect the overall estimate for the impact of probiotic/synbiotic supplementation on the length of stay in ICU (CI: −5.453, 0.912). Furthermore, the funnel plot supports the absence of a publication bias (Figure 5(b)). The Egger test also confirms the absence of publication bias. There was no evidence of publication bias using Egger's test (*P*=0.071). The GRADE system was used to calculate the quality of evidence for the length of stay in the ICU (based on inconsistency and imprecision) (Table 3).

## 4. Discussion

This study systematically reviewed seven RCTs to investigate the impacts of probiotics/synbiotics supplements on inflammatory biomarkers (serum/plasma levels of CRP and IL-6) and the length of ICU stay among trauma and traumatic brain injury patients. The pooled analysis indicated that probiotics/synbiotics supplementation did not affect serum/plasma CRP levels significantly compared with control groups. Supplementation with probiotics and synbiotics had no effect on serum or plasma IL-6 concentrations or the length of ICU stays. Our hypotheses about reduced levels of CRP, IL-6, and length of ICU stays were not fully substantiated.

There was evidence of high heterogeneity in the data. We performed subgroup and sensitivity analyses to find the sources of heterogeneity. In terms of CRP and IL-6, the heterogeneity seemed to be explained by one study [30], and when this was excluded, there was no evidence of heterogeneity. It should be noted that this study was conducted on young people. The effect of probiotics on severe inflammation in younger ages might be explained by dysregulated inflammatory pathways caused by age-related obesity. In the elderly, chronic low-grade inflammation may impair the therapeutic effects of probiotic consumption [52].

The stress response after trauma has been extensively studied, and it includes significant electrolytic, hormonal, and metabolic changes, as well as cytokine release [53, 54]. The stress reaction causes splanchnic vasoconstriction, leading to hypoxia and ischemia in the gut tissue after severe trauma [55]. The gut microbiota regulates the immune system by producing molecules that have immune-modulatory and anti-inflammatory activities and stimulate immune cells [56].

Nutritional support is a crucial issue in intensive care units, and using some compounds with anti-inflammatory and antioxidant activators is thought to be beneficial for critically ill patients [16]. Probiotics can help prevent or repair “perforated” epithelial barriers as well as influence the inflammatory response indirectly by counteracting the source of the pro-inflammatory stimulus associated with low endotoxemia [57]. According to recent clinical data, the administration of probiotics can decrease ICU infections [23].

CRP is a clinically important hepatic-derivedacute-phase protein that rises in response to IL-6 secretion by T cells and macrophages [58, 59]. CRP levels increased in patients with multiple injuries in the early period after trauma, especially on the second day. Therefore, monitoring CRP parameters could be useful to recognize patients who are susceptible to infection in the first two days after hospitalization [60]. Our findings show that probiotic/synbiotic supplementation had no effect on serum/plasma CRP levels in both short and long intervention periods (i.e., <21 days and ≥21 days). However, in the mean age subgroup analysis, a reduced serum/plasma level of CRP was observed among those aged over 40 years. A chronic inflammatory condition which cannot be sufficiently resolved or restrained arises primarily as individuals age. On the other hand, trauma aggravates inflammatory conditions, and the immune system appears to be unable to control the inflammatory response punctually [61]. As a result, CRP reduction may be attributed to the elevated CRP levels and, therefore, is effective in patients aged >40 years compared with those aged <40 years.

IL-6 is a prototypical cytokine for preserving homeostasis. When homeostasis is disturbed after infection or tissue damage, IL-6 is instantly produced to assist the host's defense against such stress by stimulating acute-phase and immunological responses [62]. The effect of probiotic/synbiotic supplementation in three studies on serum/plasma IL-6 concentration was not significant in the present meta-analysis. However, only Wan et al. reported a remarkable reduction of IL-6 levels in patients with severe traumatic brain injury. These positive results may be attributed to the relatively large sample size (i.e., including 76 patients). Because serum/plasma CRP was significantly reduced in our meta-analysis and IL-6 is also the main activator of CRP [60], it appears that additional studies with a sufficient reasonable sample size should be performed to find serum/plasma CRP and IL-6 associations with probiotic/synbiotic supplementation.

Due to immunological disturbance following a severe traumatic injury, it seems that patients are more vulnerable to nosocomial infections, resulting in an extended length of ICU stay [32]. The meta-analysis carried out by Gu et al. analyzed trauma outcomes with two studies and reported that the length of ICU stay was reduced with probiotic supplementation [63]. However, despite including further studies, the length of ICU stay was not significantly reduced in our meta-analysis. The Glasgow Coma Scale is a crucial measure to classify trauma severity. The usual standard for determining whether a head injury is severe or moderate to mild is the Glasgow Coma Scale (GCS) ≤ 8 [64]. The management of patients is commonly dependent on GCS categorization [64]. Due to the low GCS (less than 8) in some studies, probiotic supplementation may improve GCS and reduce the length of ICU stay [30, 46, 51].

Probiotic consumption may reduce inflammatory biomarkers by increasing short-chain fatty acid (SCFA) production in the gut [65]. SCFA may quench the synthesis of hepatic CRP and result in reduced inflammation and oxidative stress [66]. Probiotics also increase the production of antimicrobial peptides, which impact the mucosa's inflammation resolution pathways and promote the production of several regulatory cytokines by stimulating the development and activity of immune cells such as dendritic cells and T cells [67, 68]. Butyrate, as an SCFA, influences pathways inhibiting NF-kB-induced increases in pro-inflammatory cytokines through some mechanisms [69]. It has also been proposed that probiotic administration reduces inflammation and oxidative stress by raising glutathione levels, hydroxyl radicals, and scavenging superoxide, thereby reducing IL-6 production in adipocytes [70, 71]. Furthermore, probiotics are recognized for their ability to stimulate adenosine monophosphate (AMP) production and activity. Probiotics such as lactobacillus fermentum have been shown to stimulate the production of AMP human beta-defensin-2 via pro-inflammatory pathways including the transcription factors NF-kB and activator protein (AP)-1, as well as mitogen-activated protein kinase (MAPK). In addition, probiotics may be able to assist in modulating the inflammatory response indirectly by increasing AMP production and secretion [72]. In addition, the binding of probiotics and microflora to innate immune system receptors such as toll-like receptors (TLRs) and Nod-like receptors (NLRs) helps regulate major intracellular pathways, which in turn helps maintain a homeostatic balance between anti-inflammatory and anti-inflammatory reactions at mucosal surfaces [69]. The major strength of the present study is that it is the first to investigate the effects of probiotic supplementation on CRP, IL-6, and the length of ICU stay in traumatic brain injuries and multiple traumas patients as a systematic review and meta-analysis of randomized controlled trials. Moreover, some limitations need to be noted. Due to the many strains and dosages of probiotics, we could not assess the effect of a specific probiotic strain and its dosage on trauma patients. It appears that more RCTs are needed to distinguish the alteration mechanism of inflammatory biomarkers by specific probiotic strains with proper dosages. Due to fewer studies, we could not evaluate the results of the inflammatory factor interleukin-6 in different subgroups. Furthermore, a protocol has not been preregistered for this review.

## 5. Conclusion

The main result of this meta-analysis was that probiotic/synbiotic supplementation did not affect serum/plasma CRP and IL-6 levels or the length of stay in the ICU. Although it should be noted that after performing the subsequent subgroup analysis of CRP based on age and intervention duration, it reveals that probiotic/synbiotic supplementation in the >45-yearold subgroup and both durations (a subgroup of >21 and ≤21 days) showed beneficial impacts. More comprehensive, well-designed clinical trial studies with appropriate sample sizes are needed to determine the effect of probiotic/synbiotic on IL-6 and the length of stay in the ICU in patients with traumatic brain injuries and multiple traumas.

## Figures and Tables

**Table 1:** Characteristics of the included studies.

Author, year	Country	Participants (intervention, control)	Age (intervention, control, yrs.)	Duration (days)	Intervention (probiotic strains)	Outcome
Kotzampassi et al., 2006	Greece	35, 30	(52.9 ± 19, 55.9 ± 18)	15	Synbiotic (contains a combination of 1011 CFU of each of four probiotics; *Pediococcus pentoseceus*, *Leuconostoc mesenteroides*, *L. paracaseissp*, and *L. plantarum*, as well as 2.5 g each of inulin, oat bran, pectin, and resistant starch)	(i) Synbiotic group compared with the control group: no significant changes in mortality (ii) Synbiotic group compared with the control group: significant changes in CRP (until 15 day), TNF-a (until 7 day), IL-6 (until 15 day) significant changes days of stay in the ICU. Synbiotic-treated group: significantly reduced in mortality
Spindler-Vesel et al., 2007	England	26, 26	48 (29.5–60), 41 (26–54)	7	Synbiotic (contains a combination of 10^10^*Pediococcus pentosaceus*, 10^10^*Lactococcus raffinolactis*, 10^10^*Lactobacillus paracasei* subsp. paracasei, 10^10^*Lactobacillus plantarum*, and 2.5 g of each of the following 4 fibers: *β* glucan, inulin, pectin, and resistant starch per sachet)	(i) Synbiotic group compared with the control group: no significant changes in CRP and stay in ICU days
Giamarellos-Bourboulis et al., 2009	Greece	36, 36	(52.9, 55.9)	15	Synbiotic 2000Forte	(i) Significant changes in CRP were observed between the two groups
Tan et al., 2011	China	26, 26	(40.5 ± 13.0, 40.8 ± 12.8)	21	Probiotic (included 0.5 × 10^8^*Bifidobacterium longum*, 0.5 × 10^7^*Lactobacillus bulgaricus*, and 0.5 × 10^7^*Streptococcus thermophiles*)	(i) Probiotics significantly increased in IL-12p70 and IFN*γ* compared with controls (ii) Probiotics significantly decreased in IL-4, IL-10, IL-6 and CRP compared with controls
Habib et al., 2020	Egypt	32, 33	(39.08 ± 7.11, 39.88 ± 7.90)	9.46	Probiotic (Lacteol Forte® sachet)	(i) Probiotic group compared with the control group: no significant changes in mortality and days of stay in the ICU
Wan et al., 2020	China	38, 38	(35.97 ± 13.12, 38.65 ± 11.26)	15	Probiotic (*Bifidobacterium longum*, *Lactobacillus bulgaricus*, and *Enterococcus faecalis* >1.0^*∗*^10^7^ CFU)	(i) Probiotic group compared with the control group: no significant changes in mortality (ii) Significant changes in days of stay in the ICU (iii) IL-6, IL-10, TNF-a, and CRP at 7 and 15 days were significantly decreased
Brenner et al., 2020	The United States	16, 15	(37.9 ± 38.5, 36.7 ± 6.2)	56	*L. reuteri* DSM 17938 drops, 100 million CFU	(i) Probiotic group compared with the control group: no significant changes in CRP, IL-1*α*, IL-1*β*, IL-2, IL-6, IL-8, IL-10, IL-12p70, TNF*α*, and IFN*γ*

CRP, C-reactive protein; ICU, intensive care unit; IL, interleukin; TNF-a, tumor necrosis factor-alpha; CFU, colony forming unit; IFN*γ*; interferon gamma.

**Table 2:** Pooled estimates of probiotic/synbiotic effects on CRP and length of stay in ICU within different subgroups.

Group	No. of comparisons	SMD (95% CI)	*P* value	*I * ^2^ (%)	*P* heterogeneity
CRP					
Intervention duration (day)					
<21	3	−1.384 (−2.670, −0.097)	0.035	93.8	≤0.001
≥21	2	−0.722 (−1.166, −0.277)	0.001	0	0.785
Mean age (year)					
>40	3	−0.742 (−1.061, −0.423)	≤0.001	7.2	0.340
≤40	2	−1.720 (−3.817, 0.378)	0.108	94.7	≤0.001
Supplement type					
Multistrain probiotic	2	−1.771 (−3.743, 0.202)	0.078	95.4	≤0.001
Synbiotic	2	−0.716 (−1.254, −0.178)	0.009	53.4	0.143
Length of stay in the ICU					
Mean age (year)					
≥40	2	−0.313 (−1.206, 0.581)	0.493	82.7	0.016
<40	2	−0.094 (−1.178, 0.990)	0.866	90.3	0.001
Supplement type					
Multistrain	3	−0.280 (−1.020, 0.460)	0.458	84.6	0.002
Synbiotic	2	−0.313 (−1.206, 0.581)	0.493	82.7	0.016

CRP, C-reactive protein; ICU, intensive care unit; SMD, standard mean difference.

**Table 3:** Summary of findings and quality of evidence assessment using the grade approach.

Measures	Summary of findings		Quality of evidence assessment (grade)						
	No of patients (trials)	Effect size ^*∗*^(95% CI)	Risk of bias^a^	Inconsistency^b^	Indirectness^c^		Imprecision^d^	Publication bias^e^	Quality of evidence^f^
CRP	283 (5)	−1.10 (−2.27, 0.06)	Not serious	Serious		Not serious	Not serious	Not serious	Moderate
IL-6	159 (3)	−2.46 (−12.16, 7.25)	Not serious	Serious		Not serious	Serious	Not serious	Low
Stay in ICU	310 (5)	−0.29 (−0.98, 0.40)	Not serious	Serious		Not serious	Serious	Not serious	Low

CRP, C-reactive protein; ICU, intensive care unit; IL-6; interleukin-6. Presented as standard mean difference (SMD). ^a^Risk of bias according to the Cochrane risk of bias tool. This tool evaluates selection bias, performance bias, detection bias, attrition bias, and reporting bias. ^b^Inconsistency (high heterogeneity). ^c^If there were factors present related to the participants, interventions, or findings that constrained the generalizability of the results, the grade would be downgraded. ^d^Large confidence interval. ^e^Downgraded if there was evidence of publication bias using a funnel plot that affected overall results detecting by trim and fill analysis. ^f^The confidence of the evidence was graded as high for all outcomes since all included studies were randomized controlled trials, and it was then downgraded following predetermined criteria. Quality was graded as high, moderate, low, and very low.

## Abbreviations

AMP: Adenosine monophosphate
CRP: C-reactive protein
GCS: Glasgow coma scale
IgA: Immunoglobulin
ICU: Intensive care unit
IL-6: Interleukin
MAPK: Mitogen-activated protein kinase
NLRs: Nod-like receptors
NF-kB: Nuclear factor kappa B
PRISMA: Preferred reporting items for systematic reviews and meta-analysis
RCT: Randomized control trials
SCFA: Short-chain fatty acids
SD: Standard deviation
SE: Standard error
SMD: Standard mean difference
TLRs: Toll-like receptors
TNF-a: Tumor necrosis factor-alpha
WMD: Weighted mean difference
SIRS: Systemic inflammatory response syndrome.

## Supplementary Materials

Supplementary Materials Supplementary Table 1. Search strategies, including the key terms and the queries for each database.
