# Supplementary material for: Effects of Probiotics Supplementation on CRP, IL-6, and Length of ICU Stay in Traumatic Brain Injuries and Multiple Trauma Patients: A Systematic Review and Meta-Analysis of Randomized Controlled Trials
Source: Evid Based Complement Alternat Med. 2022 Dec 5;2022:4674000. doi: 10.1155/2022/4674000 (PMC9744609; doi:10.1155/2022/4674000)
Supplement: Supplementary Materials — Supplementary Table 1. Search strategies, including the key terms and the queries for each database. [file 4674000.f1.docx]

**Supplemental Table 1:** Medical subject headings (MeSH) and non-MeSH terms used to search relevant publications on the relation between probiotics and traumatic injuries^1^

| Database | Step | Terms | Results |
| --- | --- | --- | --- |
| PubMed | 1 | **((((("Probiotics"[Mesh]) OR "Lactobacillus"[Mesh]) OR "Bifidobacterium"[Mesh]) OR "Streptococcus"[Mesh]) OR "Saccharomyces"[Mesh]) OR ((((((Probiotic*[Title/Abstract]) OR (Lactobacillus*[Title/Abstract])) OR (Bifidobacterium*[Title/Abstract])) OR (Streptococcus*[Title/Abstract])) OR (Saccharomyces*[Title/Abstract])) OR ("VSL#3"[Title/Abstract]))** |  |
|  | 2 | **((((("Wounds and Injuries"[Mesh]) OR "Spinal Cord Injuries"[Mesh]) OR ( "Head Injuries, Closed"[Mesh] OR "Brain Injuries, Traumatic"[Mesh] )) OR "Burns"[Mesh]) OR "Fractures, Bone"[Mesh]) OR ((((((((injur*[Title/Abstract]) OR (trauma*[Title/Abstract])) OR (Wound*[Title/Abstract])) OR (burn*[Title/Abstract])) OR (fracture*[Title/Abstract])) OR ("TBI"[Title/Abstract])) OR (polytrauma*[Title/Abstract])) OR (accident*[Title/Abstract]))** |  |
|  | 3 | **(((randomize clinical trial[Publication Type]) OR ("controlled clinical trial"[Publication Type])) OR (trial*[Title/Abstract])) OR ("clinical trial*"[Title/Abstract]) OR (supplementation[Title/Abstract]) OR (group*[Title/Abstract]) OR (random*[Title/Abstract]) OR (placebo[Title/Abstract]) OR ("randomized clinical trial"[Title/Abstract]) OR ("controlled clinical trial"[Title/Abstract])** |  |
|  | 4 | #1 AND #2 AND #3 | 1183 |
| Scopus | 1 | (TITLE-ABS-KEY (probiotic*** OR lactobacillus*** OR bifidobacterium*** OR streptococcus*** OR saccharomyces***)) |  |
|  | 2 | (TITLE-ABS-KEY (trauma*** OR injur*** OR *wound** OR *burn** OR *fracture**)) |  |
|  | 3 | (TITLE-ABS-KEY (trial*** OR clinical AND *trial** OR *supplementation* OR *group** OR *random** OR *placebo* OR *"randomized clinical trial"* OR *"controlled clinical trial”)*) |  |
|  | 4 | #1 AND #2 AND #3 | 3088 |
| Embase | 1 | probiotic*: ab,ti OR lactobacillus*:ab,ti OR bifidobacterium*:ab,ti OR streptococcus*:ab,ti OR saccharomyces*:ab,ti |  |
|  | 2 | trauma*: ab,ti OR injur*:ab,ti OR wound*:ab,ti OR burn*:ab,ti OR fracture*:ab,ti |  |
|  | 3 | trial*: ab,ti OR 'clinical trial*':ab,ti OR supplementation:ab,ti OR group*:ab,ti OR random*:ab,ti OR placebo:ab,ti OR 'randomized clinical trial':ab,ti OR 'controlled clinical trial':ab,ti |  |
|  | 4 | #1 AND #2 AND #3 | 948 |
| Total |  |  | 5219 |

^1^ Two investigators (NN & MH) searched the online databases independently.
